# Molecular Mechanisms and Therapeutics for SBMA/Kennedy’s Disease

**DOI:** 10.1007/s13311-019-00790-9

**Published:** 2019-11-04

**Authors:** Frederick J. Arnold, Diane E. Merry

**Affiliations:** Department of Biochemistry and Molecular Biology, Thomas Jefferson University, 411E Jefferson Alumni Hall, 1020 Locust Street, Philadelphia, Pennsylvania 19107 USA

**Keywords:** Polyglutamine, androgen receptor, neurodegenerative disease, motor neuron, spinal and bulbar muscular atrophy.

## Abstract

Spinal and bulbar muscular atrophy (SBMA) is a neuromuscular disease caused by a polyglutamine (polyQ) expansion in the androgen receptor (AR). Despite the fact that the monogenic cause of SBMA has been known for nearly 3 decades, there is no effective treatment for this disease, underscoring the complexity of the pathogenic mechanisms that lead to a loss of motor neurons and muscle in SBMA patients. In the current review, we provide an overview of the system-wide clinical features of SBMA, summarize the structure and function of the AR, discuss both gain-of-function and loss-of-function mechanisms of toxicity caused by polyQ-expanded AR, and describe the cell and animal models utilized in the study of SBMA. Additionally, we summarize previously conducted clinical trials which, despite being based on positive results from preclinical studies, proved to be largely ineffective in the treatment of SBMA; nonetheless, these studies provide important insights as researchers develop the next generation of therapies.

## History and Background

Progressive proximal spinal and bulbar muscular atrophy (SBMA) was first described by Dr. William R. Kennedy et al. in 1968 following the examination of 2 families in which 11 members, all male, presented with a late onset, slowly progressive neuromuscular disorder [1]. Decades later, the genetic basis for SBMA was identified as the expansion of a polymorphic tandem CAG repeat within the first exon of the androgen receptor (AR) gene [2]. SBMA, the first known CAG trinucleotide repeat disorder, now exists within a disease family that includes Huntington’s disease (HD), dentatorubropallidoluysian atrophy (DRPLA), and spinocerebellar ataxia (SCA) types 1, 2, 3, 6, 7, and 17.

## Clinical Manifestations

**Fig. 1:**
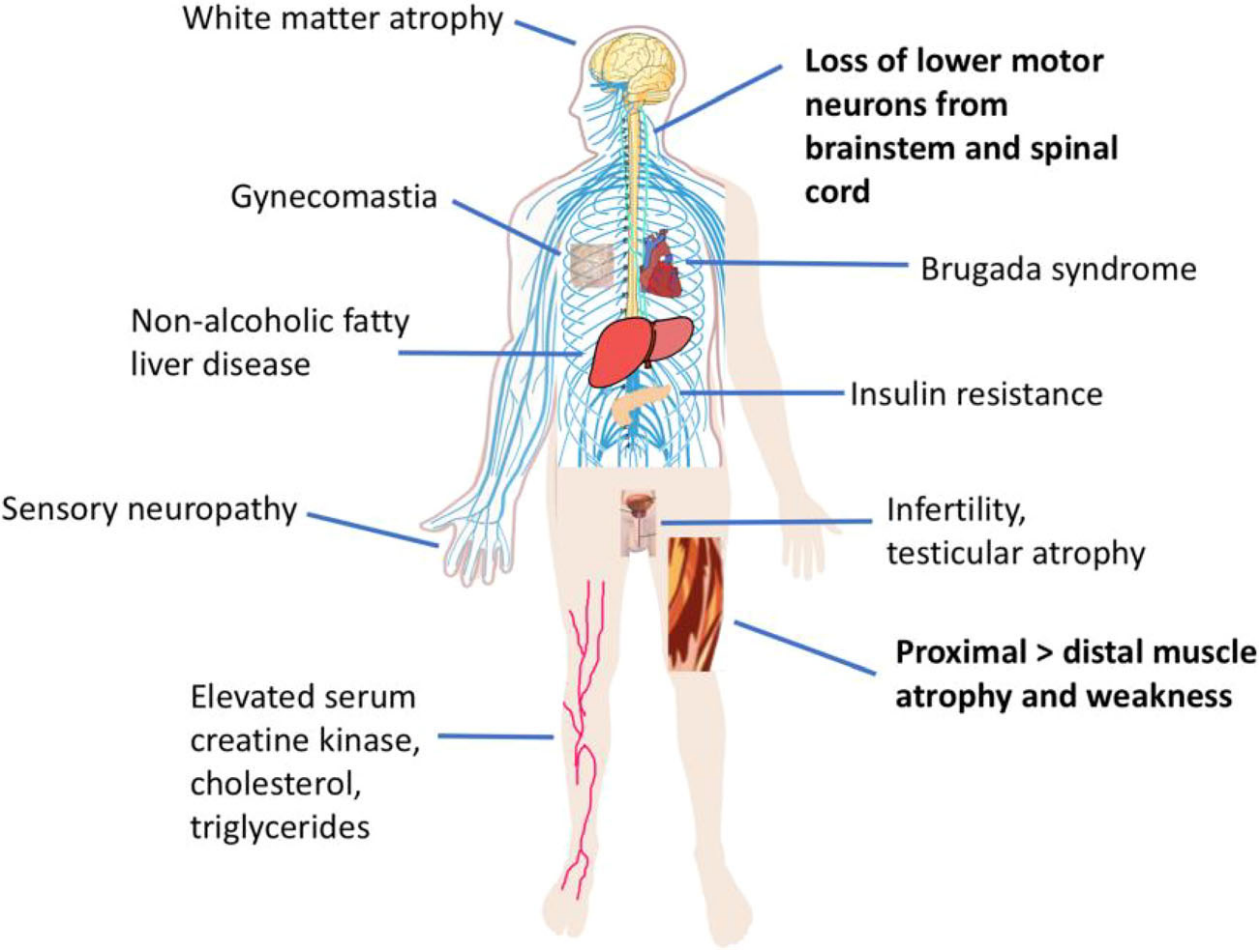
Clinical manifestations of SBMA. Although neuromuscular dysfunction (bold) is the primary clinical feature of SBMA, system-wide disturbances have been reported. Symptoms related to androgen insensitivity, altered metabolism, and sensory neuropathy also affect the quality of life of SBMA patients

The clinical manifestations of SBMA are summarized in Fig. 1. The majority of SBMA patients initially present with proximal lower limb weakness, with symptom onset typically occurring in men between 30 and 50 years of age [1, 3]. Muscle strength declines slowly, at a rate of approximately 2% per year [4], with additional symptoms appearing over time, including tremor, muscle cramps, fasciculations, dysarthria, and dysphagia [3]. Degeneration of the bulbar musculature predisposes patients to potentially fatal aspiration-induced pneumonia, which was identified as the leading cause of death in a natural history study of SBMA [3]. The predominance of neuromuscular symptoms in SBMA patients corresponds with disease pathology, which is characterized by a loss of lower motor neurons in the anterior horn of the spinal cord and in the brainstem [5, 6], as well as by signs of cell-autonomous toxicity in muscle [7–9]. Evidence of both neurogenic and myogenic abnormalities are observed in patient muscle, including atrophic and morphologically abnormal muscle fibers, fiber-type grouping, and centralized nuclei [5, 8, 10]. Indeed, the best-characterized metabolic indicator of SBMA is serum creatine kinase (CK), which is elevated to approximately 3 to 4 times the normal range in the majority of SBMA patients [11, 12]. This is higher than would be expected for a purely neurogenic disease, underscoring the fact that primary myopathy contributes to SBMA [9].

In addition to neuromuscular symptoms, SBMA patients also present with signs of androgen insensitivity, such as gynecomastia, infertility, and testicular atrophy [3, 13–15]; however, the neurologic symptoms of SBMA are not caused by a loss of AR function, as these are not observed in patients with complete androgen insensitivity syndrome [16]. Androgen insensitivity is also the presumed cause of the urinary tract symptoms experienced by some SBMA patients, as low levels of androgens have been associated with bladder outlet obstruction [12, 17] and the motor neurons that innervate the bulbospongiosus muscle do not undergo neurodegeneration [18]. It should be noted that this does not rule out cell-autonomous dysfunction of pelvic floor muscles as a contributing factor in urinary tract symptoms [12].

Numerous studies have also noted electrophysiological dysfunction and axon loss in the sensory neurons of SBMA patients, highlighting the fact that disease pathology extends beyond the neuromuscular system [5, 19–27]. Sensory neuron disruption causes a number of symptoms in patients, including vibratory sensation [28], neuropathic pain [28, 29], and numbness [30, 31]. Because these sensory symptoms do not affect quality of life to the same extent as motor symptoms in most patients, the involvement of sensory neurons in disease has not received focused attention despite being a common feature in the manifestation of SBMA.

Metabolic disturbances have also been reported in SBMA patients, with a number of recent studies greatly expanding our understanding of and appreciation for the system-wide effects of mutant AR. For example, impaired glucose homeostasis is now understood to be a common feature of SBMA [12, 32, 33]. Although the results of these studies offer conflicting evidence for whether fasting blood glucose levels are normal [14], low [33], or high [12, 32, 34] in SBMA patients, there seems to be consensus regarding an increase in insulin resistance in SBMA patients, as measured by the homeostasis model assessment of insulin resistance (HOMO-IR) [32–34]. In fact, a recent study found a significant correlation between insulin resistance and motor dysfunction in a cohort of SBMA patients [33]. Although reduced insulin receptor protein levels in skeletal muscle appear to be one contributing factor, the mechanism regarding insulin resistance in SBMA patients requires further elucidation [33]. Regardless, these results suggest that signs of metabolic disruption in SBMA patients may become a useful tool in predicting motor function impairment. Dysregulation of other metabolic indicators have also been reported in a subset of SBMA patients, including low body mass index, bone density, and HDL, and high blood pressure, total cholesterol, and triglycerides [12, 14, 33]. Moreover, SBMA patients are at a high risk for nonalcoholic fatty liver disease [32]. Interestingly, hepatic AR-knockout mice similarly develop insulin resistance, indicating that loss of AR function in SBMA patients may contribute to this phenotype [35].

As the number of known clinical presentations of SBMA continuously expands, it is worth noting that even rarely reported symptoms, when taken together, provide clues for the fundamental mechanisms of cellular toxicity that contribute to SBMA. Although not considered a primary aspect of disease manifestation, some studies have noted subtle cognitive impairment in SBMA patients, finding diminished working memory and executive function [36, 37]. Moreover, a neuropsychological analysis of 20 SBMA patients found a mentalizing deficiency, as determined by performance on the Faux Pas Test [38]. These findings suggest the involvement of the frontal lobe in SBMA pathogenesis, which is further supported by the fact that SBMA patients have glucose hypometabolism [39] and extensive atrophy of white matter in frontal areas of the brain [40]. More recently, however, a study in a larger cohort of SBMA patients (64 patients) found no impairment in neuropsychological performance [41]. Indeed, SBMA patients scored significantly *higher* in the Prose Memory test than controls, suggesting that earlier findings are not reproduced in a larger cohort and, thus, likely do not represent substantive changes [41].

Additionally, although there is no evidence of cardiomyopathy in SBMA patients [42], Brugada syndrome and other electrocardiogram (ECG) abnormalities have been identified, indicating myocardial involvement in disease which, if undetected, can lead to sudden death [12, 43]. Other rarely reported symptoms include poor sleep quality [44], jaw drop [45], selective bulbar dysfunction [46], and myotonia [43]. The heterogeneity in the clinical presentation of SBMA, a monogenic disease, suggests that environmental factors and genetic modifiers greatly shape patient phenotype.

Unique symptoms are also associated with a highly expanded CAG repeat. In a patient with 68 CAGs (currently the longest repeat identified in an SBMA patient), disease onset occurred at the age of 18, and by age 29, symptoms included autonomic nervous system dysfunction and abnormal sexual development [47]. Taken together, it is clear that SBMA affects a wide range of cell types and tissues and that, although motor neuron degeneration and neuromuscular disruption are considered hallmarks of the disease, a wider view of SBMA pathogenesis may yield new therapeutic opportunities and mechanistic insights.

Although a highly expanded CAG repeat length may be associated with additional symptoms [47], there is extensive evidence demonstrating that in SBMA, CAG repeat length inversely correlates with the age of symptom onset but not with the rate of disease progression [3, 30, 31, 48–50]. Although one study posited that a longer CAG repeat (greater than 47) is associated with a motor-dominant phenotype and a shorter CAG repeat (less than or equal to 47) is associated with a sensory-dominant phenotype (as determined by measuring compound muscle and sensory nerve action potentials) [28], a follow-up study that controlled for patient age at examination reproduced only the negative correlation between CAG repeat length and compound muscle action potential, finding no correlation between CAG repeat length and sensory nerve action potential [51]. In patient tissue, CAG repeat length directly correlates with the frequency of nuclear aggregates of polyQ-expanded AR in motor neurons [28]. Taken together, it appears that in the typical patient population, CAG repeat length correlates with age of symptom onset and AR aggregation, but not with the rate of disease progression nor with any particular symptoms of the disease. However, the characterization of additional, unique symptoms in an SBMA patient with a CAG repeat length of 68 leaves open the possibility that CAG repeat length can influence the severity of disease.

## Androgen Receptor

### Androgen Receptor Structure and Functional Domains

**Fig. 2:**
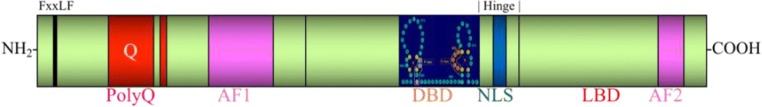
Schematic of the androgen receptor. Schematic representation of the androgen receptor (AR), depicting key functional domains of the protein. Shown here are the size and location of the DNA-binding domain (DBD) from amino acids 539 to 627, the hinge domain from amino acids 628 to 670, and the ligand-binding domain (LBD) from amino acids 671 to 919. Additionally, the polymorphic polyglutamine tract (polyQ) is depicted in the NTD along with the FxxLF motif, which participates in AR intra- and intermolecular amino-carboxyl (N/C) terminal interactions. The nuclear localization signal (NLS) is shown spanning amino acids 617 to 634 in the DBD and hinge regions. Also depicted are the two transactivation domains of the AR: the activation function 1 (AF-1) domain in the NTD and the activation function 2 (AF-2) domain in the LBD

The androgen receptor gene (GenBank: M20132.1) is located on the X chromosome at position q11-12 and contains 8 exons spanning more than 90 kb, encoding a 919 amino acid protein (Fig. 2). Along with the estrogen receptor (α and β), the progesterone receptor, the mineralocorticoid receptor, and the glucocorticoid receptor, the AR is a member of the steroid hormone receptor subfamily of the nuclear receptor superfamily. Like other nuclear receptors, of which there are at least 49 in the human genome [52], the androgen receptor consists of 4 defined domains: an amino-terminal domain (NTD), a highly conserved DNA-binding domain (DBD), a hinge domain, and a carboxyl-terminal ligand-binding domain (LBD) [53].

The length, sequence, and structure of the NTD vary between the steroid hormone receptors but typically contain at least one transactivation domain, referred to as the activation function 1 (AF-1) domain. The largest domain of the AR, the NTD spans amino acids 1 to 538 and contains a polymorphic polyglutamine tract as well as polyproline and polyglycine tracts. Although the AR NTD is intrinsically disordered in the absence of binding partners [54], over 150 coregulators are known to bind the AR NTD and coactivator binding can promote structure in the NTD [55, 56]. Additionally, the AR NTD contains an FxxLF motif (^23^FQNLF^27^) that participates in intra- and intermolecular amino-carboxyl (N/C) terminal interactions via charge-clamp hydrogen bonding with residues within the LBD [57, 58]. Recently, the first 36 amino acids of the AR NTD were identified as a putative mitochondrial localization sequence (MLS) [59]. The discovery of a bona fide MLS in the AR NTD adds to prior evidence that both wild-type and mutant AR localize to mitochondria [59–61].

The AR DNA-binding domain extends from amino acids 539 to 627 and contains two zinc finger domains composed of 3 α-helices [62]. Within the first zinc finger domain, a P-box comprised of 5 amino acids (residues 577-581) interacts with the major groove of DNA at androgen response elements (AREs) [63]. Consensus AREs consist of 2 hexameric, palindromic motifs spaced by 3 base pairs (AGAACAnnnTGTTCT) [64, 65]. The second zinc finger in the AR DBD contains a 5-amino-acid sequence referred to as the dimerization box (D-box) (residues 596-600), which, in part, mediates AR homodimerization [66]. AR binds to DNA as a dimer, with each member of the dimer binding half of the ARE [62, 67]. Like the progesterone receptor and the glucocorticoid receptor, the AR binds to canonical AREs in a ‘head-to-head’ orientation, although it may bind to noncanonical AREs as a head-to-tail homodimer [62]. Although AR homodimerization precedes DNA binding, populations of both dimerized and monomeric AR can be detected in the nucleus [66]. Intriguingly, FRET analysis demonstrated that the AR N/C interaction occurs predominantly between AR molecules of the mobile nuclear population, suggesting that DNA binding abolishes the N/C interaction, possibly allowing coactivator binding to the AF-2 domain in the AR LBD [68].

The hinge domain of the AR (residues 628-670) is so-called because it serves as a flexible bridge between the DBD and LBD. Although this region is poorly conserved, it nonetheless serves a number of important functions. A bipartite nuclear localization signal (NLS) (^617^RKCYEAGMTLGARKLKKL^634^), encompassing the end of the DBD and the first 8 residues of the hinge region, regulates AR nuclear import [69]. Binding of the AR to the importin α–importin β complex is mediated by the basic residues within ^629^RKLKKL^634^, as mutation of K630, K632, or K633 reduces this interaction and mutation of all 3 lysines completely prevents the interaction [70]. Beyond their role in AR nuclear import, the first 12 amino acids of the hinge region, referred to as the carboxyl-terminal extension of the DBD, are also involved in DNA binding, specifically in mediating the selectivity of AR transactivation at canonical *versus* noncanonical AREs [71]. Importantly, the role of residues 627 to 634 in nuclear import and DNA binding are not mutually exclusive, as the interaction between the AR and importin α dissociates in the nucleus prior to DNA binding [72]. Given their role in mediating AR nuclear localization, it is perhaps surprising that deletion of amino acids 629 to 634 results in an increase in AR transcriptional activity on some reporters despite a dramatic reduction in AR nuclear import [73]. Although coactivator recruitment is not significantly affected by this deletion, the N/C interaction was markedly enhanced [73]. Subsequent studies have further elucidated the mechanism by which deletion of 629 to 634 increases AR transactivation based on the understanding that, in the nucleus, AR molecules exist in both a mobile fraction and an immobile fraction, which is believed to be DNA-bound (this is supported by the fact that DNA-binding mutants exist in a mobile fraction) [68, 74]. Mutation of residues 629 to 630 as well as 632 to 633 greatly reduces the immobile fraction of the AR and correspondingly decreases its residence time on DNA by ~10-fold [75]. Thus, despite reducing AR nuclear import, deletion of 629 to 634 may increase AR transcriptional activity by increasing the rate at which AR cycles on DNA (a mechanism demonstrated for the estrogen receptor) [72, 76]. Moreover, the fact that mutations in the hinge region are associated with prostate cancer and increase AR transcriptional activity supports the notion that the hinge region plays a key regulatory (predominantly inhibitory) role in AR activity [73]. There is also evidence that the hinge domain plays a role in facilitating the intramolecular N/C interaction, as overexpression of sirtuin 1 (SIRT1), which deacetylates the AR at K630, K632, and K633, reduces the intramolecular N/C interaction [77].

The C-terminal LBD of the AR, spanning amino acids 671 to 919, is modestly conserved in terms of sequence, but its 12 α-helix secondary structure is well-conserved [78]. Upon hormone binding, the position of helix 12 shifts, covering the ligand binding pocket (composed of the other 11 α-helices) and exposing the AF-2 domain to coactivator binding [58, 79]. Antagonist binding to steroid hormone receptors causes helix 12 to reposition differently, blocking the binding of some coactivators and/or recruiting the binding of corepressors [80]. As previously described, the AF-2 domain of the AR LBD binds to the ^23^FQNLF^27^ motif in the NTD via both intra- and intermolecular interactions. Temporally, hormone binding in the cytoplasm induces an intramolecular N/C interaction, which is followed by intermolecular N/C interactions in the nucleus [66]. This interaction is essential for the transactivation of certain genes (one notable exception being the mouse mammary tumor virus (MMTV)) [81]. Additionally, recent work revealed that the AR LBD can homodimerize in a head-to-head conformation, with the interface of AR monomers centered around helix 5 [82]. Perhaps because the primary interactor with the AF-2 domain is the FxxLF motif in the NTD, interactions between coactivators and the AR AF-2 domain are typically weaker than those formed with the AF-1 domain [83]. Thus, the AF-2 domain plays a diminished role in AR transcriptional activity compared with other steroid hormone receptors [84]. Mutagenesis experiments have identified an additional role for the LBD in AR nuclear export, as a 75-amino-acid region (residues 743-817) is both necessary and sufficient for AR nuclear export in a PC3 prostate cancer cell model [85]. The putative nuclear export signal (NES) contained within this region is leptomycin B-insensitive, suggesting that this regulatory motif is not a candidate for chromosome region maintenance 1 (CRM-1)–mediated nuclear export upon hormone withdrawal [85–87].

### Cellular Trafficking

Prior to ligand binding, the AR resides in the cytoplasm in an inactive aporeceptor complex that contains chaperones (Hsc70, Hsp40, Hsp90, HIP, HOP), p23, and immunophilins (Cyp40, FKBP51, FKBP52) [88, 89]. The aporeceptor complex maintains the AR in a high affinity state for hormone binding [90], whereas an association with importin 7 retains AR in the cytoplasm by blocking the interaction between importin α and the bipartite NLS of the AR [91]. It has been proposed that karyopherins such as importin α act as chaperones by binding exposed basic residues on their client proteins [92, 93]. Unlike some other steroid hormone receptors, the AR is rapidly degraded in its unliganded state [94].

Testosterone is primarily produced by Leydig cells in the testes in response to secretion of luteinizing hormone (LH) from the pituitary gland [95]. Extracellularly, most testosterone is bound by sex hormone-binding globulin (SHBG) and albumin, but the free form can passively diffuse across cell membranes [95]. In cells expressing the enzyme 5α-reductase, testosterone is converted intracellularly into the more potent androgen, dihydrotestosterone (DHT) [95, 96]. Notably, there are 3 isoenzymes of the steroid 5α-reductase (SRD5A) family, characterized by different expression patterns and kinetic properties [97, 98]. Upon binding of testosterone or DHT, the AR undergoes a conformational change, dissociating from the aporeceptor complex and importin 7 [91]. This conformational change also induces the intramolecular N/C interaction and exposes the NLS to binding by importin α, facilitating nuclear import [66, 70].

Following nuclear translocation, the AR binds to DNA, canonically as a homodimer [66], but can also be tethered to DNA through interactions with other transcriptional regulators. Coactivator binding to the AR primarily via the AF-1 domain of the NTD leads to the recruitment of RNA polymerase II at the promoter, thus enabling transcription [99].

There is evidence that the AR is exported from the nucleus and subsequently degraded in the cytoplasm by the ubiquitin–proteasome system [100]. In addition to the NES identified in the AR LBD, residues in both the DBD and the hinge region also appear to regulate AR nuclear export. A double mutation in the DBD (F582, 583A) is sufficient to block nuclear export; however, DNA binding itself is not required for AR nuclear export, as the DNA-binding mutant V581F does not affect export [101, 102]. Additionally, phosphorylation of the AR at serine 650 has been shown to regulate nuclear export of the AR [103–105]. In COS-7 and LNCaP cells, phosphorylation of S650 is mediated by the MAPK kinase 4/JNK and MAPK kinase 6/p38 stress kinase signaling pathways. Inhibition of JNK and p38 reduced the nuclear export of wild-type AR in COS-7 cells to an equivalent extent as a phospho-null (S650A) mutation but had no effect on AR with a phospho-mimic (S650D) mutation [103]. Consistent with these findings, knockdown of MAP3K11, an upstream regulator of JNK activity, decreased S650 phosphorylation in LNCaP and C2-4B cells [106]. Additionally, inhibition of protein phosphatase 1 alpha (PP1α) by tautomycin led to an increase in S650 phosphorylation and a decrease in AR nuclear accumulation in LNCaP cells [104]. Notably, increasing AR nuclear export is associated with a decrease in its transcriptional activity, whereas blocking export increases AR transcriptional activity [101, 103, 104, 107].

## Polyglutamine Expanded AR

### Structural Changes

The length of the polyQ tract in the AR NTD causes structural changes with important implications for AR function. Although the NTD is relatively unstructured in the absence of coregulator binding [56], expansion of the polyQ tract leads to an increase in α-helical structure, as determined by circular dichroism and nuclear magnetic resonance (NMR) spectroscopy [108, 109]. Correspondingly, shorter polyQ tracts exhibit reduced α-helical structure [108, 110]. As polyQ tract length increases, unconventional hydrogen bonds accumulate between glutamine side chains and main chain carbonyl groups, stabilizing α-helices [109]. Other studies have found that expanded polyQ tracts increase the propensity of a protein (including the AR) to form antiparallel β sheets [111–114]. The critical threshold for disease is approximately 40 glutamines in several of the polyQ repeat disorders, suggesting that a fundamental structural change may occur at this length. At about 40Q, the polyQ tracts of different proteins may form β-pleated sheet structures capable of self-associating into polar zippers (so-named because they resemble the leucine zipper interaction that mediates the binding of c-Jun to c-Fos) [115]. Additionally, a cylindrical β-sheet structure has been shown to be unstable at 20Q, but stable and capable of nucleating further helical growth at 40Q [116]. Relative to AR20Q, AR45Q also exhibits increased binding to the hydrophobic probe 8-anilinonaphthalene-1-sulphonic acid and increased sensitivity to urea-induced unfolding [108]. This correlates with data suggesting that the stability of AR is inversely proportional to its polyQ tract length [117]. Although an expanded polyQ tract has not been shown to alter the AR N/C interaction, blocking the N/C interaction of polyQ-expanded AR is protective in SBMA cell and animal models [118, 119]. Disruption of the N/C interaction substantially increases phosphorylation of the AR at S16, and its protective effect is dependent on S16 phosphorylation [118]. Whether the polyQ expansion affects the N/C interaction or S16 phosphorylation remains an open question; however, given that the intermolecular N/C interaction stabilizes the AR homodimer [120, 121], it may be that disrupting the N/C interaction exerts a protective effect irrespective of whether or not the polyQ expansion disrupts these processes.

#### Altered Posttranslational Modifications

The AR is known to be regulated by phosphorylation, ubiquitination, methylation, SUMOylation, palmitoylation, and acetylation, with some of these posttranslational modifications altered by the polyQ expansion. For example, reduced phosphorylation of the mutant AR at S650 may contribute to its impaired nuclear export [105]. Additionally, hyperacetylation of the AR at K630, K632, and K633 correlates with enhanced toxicity of AR with acetyl-mimic K630, K632, and K633Q/T mutations and with reduced toxicity of AR with K630, K632, and K633R mutations in cells [122]. PolyQ-expanded AR also exhibits increased arginine methylation relative to wild-type AR, due to an enhanced interaction with protein arginine methyltransferase 6 (PRMT6) [123]. Correspondingly, knockdown of PRMT6 was protective in SBMA MN-1 cells and knockdown of the *Drosophila* homolog of PRMT6 (DART8) suppressed the degenerative eye phenotype of flies expressing AR52Q [123]. Other studies have demonstrated that modulating the posttranslational modifications of the mutant AR, even in the absence of a baseline difference with wild-type AR, can be protective. For example, increasing AR phosphorylation at S215 and S792 (Akt consensus sites) reduces AR toxicity in cell and animal models of SBMA [124, 125]. Similarly, both wild-type and polyQ-expanded AR are phosphorylated at S96 by cyclin-dependent kinase 2 (CDK2), and reducing the phosphorylation of the mutant AR S96 by activation of the adenylyl cyclase (AC)/protein kinase A (PKA) signaling pathway is protective in cell and animal models of SBMA [126]. Furthermore, phosphorylation of the AR at S514 (a MAPK consensus site) blocks mutant AR-induced toxicity and caspase-3 cleavage of the AR in HEK293T and MN-hybrid cells [127]. Blocking the SUMOylation of mutant AR attenuates muscle atrophy and behavioral deficits and extends survival in a mouse model of SBMA, highlighting a protective effect of increasing AR transcriptional activity *in vivo* [128]. Taken together, these data indicate both that polyQ-expanded AR exhibits an altered posttranslational modification profile and that modulating the posttranslational modifications of the mutant AR can reduce its toxicity in SBMA cell and animal models.

#### Altered Trafficking and Function

Considering that the AR interacts with approximately 250 different proteins [129], it is unsurprising that structural changes in the AR NTD caused by a polyQ expansion can alter this interactome. For example, Pax Transactivation-domain-interaction Protein (PTIP) has been shown to interact with mutant AR, but not with wild-type AR [130]. Given the role of PTIP in DNA damage repair, it was hypothesized that this aberrant interaction could impair the ability of PTIP to perform its normal function [130]. In a similar way, retinoblastoma protein (Rb) interacts more strongly with mutant AR than with wild-type AR, inappropriately inducing E2F1 transcriptional activation in a *Drosophila* model of SBMA [131]. Cytochrome *c* oxidase subunit Vb (COXVb) interacts more strongly with soluble wild-type AR than with polyQ-expanded AR, but importantly, also colocalizes with AR aggregates [61]. This altered interaction (perhaps especially the sequestration of COXVb into AR aggregates) could contribute to the mitochondrial dysfunction observed in cell and animal models of SBMA [60, 132], as well as in SBMA patient tissue [133]. Moreover, it may be that the potential for mitochondrial localization of AR, driven in part by its mitochondrial localization signal [59], contributes to both COXVb sequestration and mitochondrial dysfunction. The extent to which the sequestration of proteins into AR inclusions contributes to proteotoxicity will be more fully addressed below.

Although polyQ-expanded AR translocates to the nucleus with comparable kinetics as wild-type AR [128, 134], both its intranuclear mobility and nuclear export rate are reduced [105]. Additionally, a number of studies have demonstrated that the length of the AR polyQ tract is inversely proportional to its transcriptional activity [135–137]. Indeed, there is evidence that increased transcriptional activity of AR with shorter polyQ tracts corresponds to an increased risk of prostate cancer [138]. The effect of polyQ tract length on AR transcriptional activity may be cell-type dependent, however, as one study found a positive correlation between polyQ length and AR transcriptional activity in C2C12 skeletal muscle cells [139]. There are several potential mechanisms by which polyQ tract length and AR transcriptional activity may be related. As discussed above, changes in the structure of the AR NTD can change the AR interactome, including interactions with coactivators [135, 140]. Additionally, it has been shown that N-terminal fragments of polyQ-expanded AR can interact with and disrupt the transcriptional activity of full-length polyQ-expanded AR [141]. Along similar lines, it is possible that accumulation of the mutant AR into small, potentially difficult-to-detect oligomers may contribute to its reduced transcriptional activity. Intriguingly, a more recent study found increased binding of AR64Q to DNA (specifically, to the MMTV promoter) despite reduced transactivation [134]. This could reflect a reduced capacity for the mutant AR to appropriately cycle on/off DNA, perhaps due to altered cofactor recruitment. Indeed, the cyclical assembly/disassembly of transcription cofactors with the estrogen receptor directly relates to its transcriptional activity [76]. The pathogenic consequences of enhanced DNA binding by polyQ-expanded AR have not been studied.

#### Aggregation

PolyQ-expanded AR exhibits not only a partial loss of its normal transcriptional function (as described above), but also a gain of new, toxic properties. Indeed, it is clear that the neurologic symptoms of SBMA are not caused by a loss of AR function, as these are not observed in patients with complete androgen insensitivity syndrome [16]. Instead, SBMA is caused by the acquisition of a toxic property or properties of the mutant AR, with aberrant aggregation likely conferring some of these toxic effects. In SBMA patients, nuclear, and to a lesser extent cytoplasmic, AR aggregates are found throughout the CNS as well as in peripheral tissues [18, 28, 142, 143]. These aggregates are morphologically granular and do not associate with membranes [143]. Mitochondria are found in proximity to AR aggregates, suggesting that the process of aggregation (and/or the maturation of aggregates) requires energy [144]. By atomic force microscopy (AFM), AR65Q was reported to form fibrils 300 to 600 nm in length, whereas AR22Q formed only annular oligomers 120 to 180 nm in diameter [145]. AR aggregation species have been further evaluated by SDS-agarose gel electrophoresis (SDS-AGE), an established method for resolving large aggregates of polyQ proteins [146–148]. By SDS-AGE, polyQ-expanded AR forms 2 distinct aggregation species. Slow-migrating aggregates contain full-length AR, are SDS-soluble, and are larger and more heterogeneous by AFM. Fast-migrating aggregates, on the other hand, contain both full-length AR and N-terminal AR fragments, are largely SDS-insoluble, and are smaller and more homogeneous by AFM [149, 150]. Additionally, slow-migrating aggregation species are detected by the 3B5H10 monoclonal antibody, which recognizes expanded polyQ tracts in low molecular weight oligomers, but not in higher molecular weight inclusion bodies [151].

Early studies of AR aggregation in SBMA patient tissue failed to detect AR aggregates with antibodies directed to the C terminus of the AR [18, 143], which was determined to be due to proteolytic cleavage of the mutant AR in a subsequent analysis in an SBMA mouse model [152]. This process was further elucidated *in vitro*, with both biochemical and imaging assays revealing that mutant AR aggregates first as a full-length protein, becoming proteolyzed over the course of inclusion maturation [150]. Moreover, soluble AR aggregates were shown to contain full-length protein [150]. Although there is no correlation between the number of AR inclusion bodies and neurodegeneration in a *Drosophila* model of SBMA [153]—in agreement with the finding that fully formed inclusion bodies of polyQ-expanded huntingtin are associated with a reduced risk of death in a cell model of HD [154]—it is clear that AR aggregation disrupts normal cellular function at least partially via the sequestration of other proteins. Proteins integrally important for normal cell function and survival have been found in AR inclusions, such as the transcriptional coactivators CREB-binding protein (CBP) and steroid receptor coactivator-1(SRC-1) [155]; the chaperones Hsp40, Hsp70, and Hsp90 [144, 156–158]; and components of the ubiquitin–proteasome system: ubiquitin, REG-gamma, NEDD8, and PA700 and 26S proteasome caps [18, 144, 159, 160]. Thus, although the extent to which soluble and/or insoluble AR aggregation species contribute to SBMA pathogenesis is uncertain, it seems likely that at least some forms of AR aggregates contribute to toxicity in SBMA.

## Modeling SBMA

### *In Vitro* Models

In the decades since the genetic cause of SBMA was identified, a number of cell models have been developed to recapitulate aspects of the disease (Table 1). Both polyQ length- and hormone-dependent toxicity are considered essential phenotypes for an SBMA model system, but the various SBMA cell lines have distinct advantages and disadvantages in mirroring other aspects of the human disease.

**Table 1:** Modeling SBMA—*in vitro* models

Cell model	Species	Cell type	PolyQ length	Aggregation	Hormone-dependent cell death	Reference
PC12	Rat	Pheochromocytoma-derived	10Q/112Q	Yes	Yes	[161]
NSC-34	Mouse	Motor neuron-derived	0Q/23Q/46Q	Yes	No	[165]
MN-1	Mouse	Motor neuron-derived	24Q/65Q/100Q	No	No	[123, 163]
C2C12	Mouse	Immortalized myoblasts	24Q/100Q	No	No	[170]
iPSC-derived NPC	Human	Neuronal precursor cells	Varied	Yes	No	[168]
Mesenchymal stem cells	Human	Adipose-derived	Varied	Yes^*^	No	[173]

SBMA cell models are further reviewed by Pennuto and Basso [225]

^*^Mesenchymal stem cells from SBMA patients form aggregates upon proteasomal inhibition via treatment with MG132

A PC12 cell model of SBMA, in which rat pheochromocytoma-derived cells express full-length human AR under the control of a tetracycline-inducible promoter, is unique among SBMA cell models in that PC12 cells readily form intranuclear inclusions of aggregated AR in a polyQ length- and hormone-dependent manner [161]. Although the role of intranuclear inclusions in SBMA pathogenesis is still debated, the inclusions formed in PC12 cells reproduce the aberrant AR proteolysis observed in SBMA patient autopsy material [18]. PC12 cells expressing AR112Q also exhibit DHT-dependent cell death, and importantly, several studies have demonstrated that a change in toxicity and/or AR aggregation in the PC12 cell model correlates with altered phenotypes in mouse models of SBMA [118, 122, 128, 162].

Another widely studied immortalized cell model of SBMA is the motor neuron-derived 1 (MN-1) model, in which AR24Q, AR65Q, or AR100Q is expressed under the control of the CMV promoter [123, 163]. These cells, originally generated by fusing embryonic mouse spinal cord motor neurons with mouse neuroblastoma cells [164], express motor neuron markers, making them a useful tool for studying the effect of polyQ-expanded AR expression in a disease-relevant cell type, as the use of primary motor neuron cultures and iPSC-derived motor neurons requires considerably more time and resources. Although these cells recapitulate the mitochondrial dysfunction that has been observed in patient tissue [60], AR65Q and AR100Q do not aggregate or cause hormone-dependent cell death in MN-1 cells.

Similarly, mouse neuroblastoma-spinal cord (NSC)-34 cells have been utilized as a motor neuron-like cell line in the study of SBMA [165]. NSC-34 cells stably expressing AR46Q exhibit cytoplasmic and perinuclear aggregates [165], potentially reflective of the cytoplasmic aggregates observed in some patient tissues [28, 142]. In the absence of hormone, AR46Q expression in NSC-34 cells decreases flux through the proteasome [166]. Testosterone treatment and subsequent AR aggregation restores proteasomal function, suggesting that sequestration of the mutant, misfolded AR into cytoplasmic aggregates preserves protein quality control in the cell [166]. These cytoplasmic AR aggregates activate autophagy in NSC-34 cells but are not properly recruited to newly formed autophagosomes, demonstrating that autophagic flux is also impaired by misfolded AR [167] (a finding replicated in other SBMA models [168]). Thus, these cells have served as a useful model in the study of protein quality control pathways in SBMA.

Corresponding with a growing recognition in the SBMA research community that muscle plays a primary role in disease pathogenesis [8, 9, 169], a muscle cell model of SBMA was developed in which C2C12 myoblasts transduced by lentivirus stably express AR24Q or AR100Q [170]. As in MN-1 cells, AR100Q does not aggregate or cause hormone-dependent cell death in C2C12 cells, although morphological analysis of these cells revealed that AR100Q expression blocks the trophic effect of DHT [170]. Treatment of C2C12 myoblasts with the β-agonist clenbuterol restored the trophic effect of DHT, which was predictive of improved muscle pathology and motor function in a mouse model of SBMA [170]. Moreover, C2C12 cells expressing AR97Q display reduced expression of the creatine transporter SLC6A8, a finding that was mirrored in SBMA patients [171].

It is important to note that in the aforementioned immortalized SBMA cell models, AR is overexpressed and contains a polyQ tract that far exceeds what is typically found in peripheral blood lymphocytes of patients (the longest patient polyQ tract length reported to date being 68Q) [47]. In order to study endogenously expressed AR in human cells, several groups have recently generated and characterized iPSC-derived neuronal cells and mesenchymal cells from SBMA patients [168, 172–174]. The study of both iPSC-derived motor neurons and iPSC-derived neuronal precursor cells (NPCs) has revealed novel SBMA phenotypes, including reduced histone deacetylase 6 activity [172] and autophagic flux defects [168], both of which were subsequently observed in patient tissue. Additionally, iPSC-derived motor neurons from SBMA patients were recently found to have defects in neurite morphology, decreased protein translation, reduced survival in prolonged culture, and dysregulation of neuronal-related signaling pathways [174]. Adipose-derived mesenchymal stem cells isolated from SBMA patients provide researchers with an additional tool, as aggregation of mutant AR can be induced in these cells by treatment with the proteasome inhibitor MG132 [173].

### *In Vivo* Models

Transgenic *Drosophila* models allow for high-throughput genetic and pharmacologic screening *in vivo*. Using the GAL4/UAS system to drive cell-type–specific expression of mutant AR, *Drosophila* models of SBMA have been shown to display hormone-dependent toxicity in photoreceptor neurons, motor neurons, and pan-neuronally [153, 175, 176]. Upon exposure to DHT, flies expressing polyQ-expanded AR exhibit both motor function deficits as well as neuromuscular junction pathology that mirrors what is observed in some mouse models [153, 175, 176]. It should be noted that *Drosophila* expressing wild-type AR also exhibit toxicity in response to DHT, although to a lesser extent than those expressing polyQ-expanded AR [123]. This is likely due to overexpression of the AR, but it nonetheless raises an important caveat in the interpretation of data derived from this model.

A number of SBMA mouse models have been generated using a variety of genetic strategies, each with a unique set of strengths and weaknesses in their ability to recapitulate the human disease (Table 2). Transgenic mice expressing AR97Q under the control of the chicken β-actin promoter with a cytomegalovirus (CMV) enhancer express high levels of the mutant AR throughout all tissues [177]. Male mice lose weight by about 6 weeks of age with onset of motor impairment by 8 weeks of age, as determined by latency to fall from an accelerating rotarod [177]. Additionally, chicken β-actin-AR97Q transgenic mice are hypoactive and develop gait abnormalities [177]. Both neuronal and muscle pathology are present in these mice, as AR aggregates are detectable throughout the CNS as well as in the muscle and in the heart [177]. Additionally, histological analyses have revealed extensive muscle fiber abnormalities in this mouse model, including fiber-type grouping and atrophic fibers as well as hypertrophic fibers with centralized nuclei [125]. The 50% survival rate of these mice is approximately 3 months [177]. Female mice display a markedly less severe phenotype, but it is notable that they do develop weight loss and motor function deficits at later time points [177].

**Table 2:** Modeling SBMA—mouse models

Mouse model	Promoter	Motor dysfunction	Decreased lifespan	Motor neuron pathology	Muscle pathology	PolyQ length dependent	Reference
Transgenic AR97Q	Chicken β-actin	Yes (8 weeks)	Yes	Yes	Yes	Yes	[177]
Transgenic AR121Q^*^	Chicken β-actin	Yes (4 weeks)	Yes	Yes	Yes	Yes	[176]
Transgenic AR112Q	Prion protein	Yes (8 weeks)	No	Yes	No	Yes	[178]
YAC transgenic AR100Q	Endogenous human	Yes (11 months)	Yes	Yes	Yes	Yes	[180]
BAC transgenic AR121Q^†^	Endogenous human	Yes (13 weeks)	Yes	Yes	Yes	Yes	[8]
Transgenic AR22Q	Human skeletal α-actin	Yes	Yes	Yes	Yes	No	[181]
Knock-in AR113Q	Endogenous mouse	Yes (8 weeks)	No^‡^	No	Yes	Yes	[185]

SBMA animal models are further reviewed by Pennuto and Basso [225]

^*^Chicken β-actin-AR121Q mice express a polyQ tract encoded by alternating CAG/CAA repeats

^†^BAC AR121Q mice contain loxP sites flanking exon 1

^‡^Urinary tract obstruction leads to early death in a subset of AR113Q knock-in mice. Mice that survive this bottleneck live a normal lifespan

Recently, a new transgenic mouse model was generated in which expression of AR121Q (encoded by alternating CAG/CAA repeats) is similarly driven by a CMV enhancer and chicken β-actin promoter, but with expression levels in the muscle comparable to endogenous mouse AR (notably, the expression level in the spinal cord is still higher than endogenous) [176]. These mice exhibit a rapid decline in body weight, rotarod performance, and grip strength beginning at 4 weeks of age and a drastically shortened lifespan, with a 50% survival rate of about 7 weeks [176]. Ubiquitin-positive aggregates of the mutant AR are detectable in the CNS as well as in skeletal muscle, and histological analyses of the muscle revealed type 1 to type 2 fiber-type switching and atrophied myofibers [176]. Disease in male mice is hormone dependent; however, female mice were not evaluated [176]. Given that these mice express 3-fold less AR in the muscle than the previously described chicken β-actin-AR97Q model [176], it is likely that the longer polyQ tract of the AR121Q (CAG/CAA) model enhances the severity of the phenotype.

A more slowly progressive phenotype is observed in the prion protein (PrP) promoter-AR112Q mouse model of SBMA [178]. In this model, AR is predominantly expressed in the CNS with some, but substantially less, expression in peripheral tissues, including muscle [118, 178]. Male PrP-AR112Q mice first display a rotarod deficit at 8 weeks of age [118] and, similar to SBMA patients, develop slowly progressive motor dysfunction but not a significantly shortened lifespan [118, 122, 162, 178]. As transgenic AR expression is low in the peripheral tissues of these mice, motor dysfunction is likely due to disrupted neuronal function in this model [178]. Additionally, intranuclear inclusions of aggregated AR can be detected in the brain and spinal cord of PrP-AR112Q mice [178]. PrP-AR112Q females display only mild behavioral deficits at later time points [178].

The fact that disease progression in the previously described transgenic mouse models is hormone dependent [177, 178] supports the notion that they are reflective of at least some aspects of the human disease. Nevertheless, it is impossible to predict the extent to which toxicity in these models is amplified (or changed) by the fact that the mutant AR is overexpressed and/or that expression is regulated by an exogenous promoter. To generate an SBMA mouse model in which expression of polyQ-expanded AR is controlled by its endogenous regulatory elements, Sopher et al. utilized a yeast artificial chromosome (YAC) containing 450 kb DNA (with the 180-kb AR gene centrally located) [179, 180]. Two YAC-SBMA lines were characterized, each expressing slightly less AR100Q than endogenous mouse AR [180]. Disease onset in the higher-expressing YAC-SBMA line (80% AR100Q expression compared with endogenous mouse AR) is marked by deficits on the hanging wire test at 11 months of age [180]. Gait abnormalities first appear at 13 months, progressing to severe hindlimb atrophy and paralysis by 17 months [180]. Although aggregated AR was not detected in the neurons of these mice, inclusions were found in astrocytes within the spinal cord and the number of motor neurons was decreased in 16-month-old mice [180]. Extensive muscle pathology is also observed in this mouse model, including atrophied and hypertrophied muscle fibers, abnormal muscle fiber morphology, centralized nuclei, and fiber-type grouping [180]. A similar mouse model was subsequently generated by Cortes et al., in which mice express AR121Q with a floxed first exon on a bacterial artificial chromosome (BAC) containing 50 kb of DNA upstream and 30 kb of DNA downstream of the AR gene [8]. These mice express AR levels similar to the YAC-SBMA model but display a substantially more severe disease phenotype, likely due to a longer AR polyQ tract length. Motor dysfunction onset is at 13 weeks and the 50% survival rate is approximately 18 weeks [8]. The primary advantage of the BAC-SBMA model is that AR expression can be inactivated by Cre-mediated excision in a cell-type–specific manner. Using this approach, it has been shown that knockdown of the AR121Q transgene from skeletal muscle alone is sufficient to cause a dramatic rescue of the disease phenotype [8].

The importance of AR expression level in generating a phenotype in mice is highlighted by the ‘myogenic model’ of SBMA. In these mice, wild-type rat AR (22Q) is highly overexpressed in skeletal muscle, with expression driven by the human skeletal α-actin promoter [181]. Myogenic SBMA mice develop progressive, hormone-dependent motor dysfunction as well as neuromuscular pathology with similarities to what is observed in other SBMA mouse models, albeit with much more rapid demise [181]. This raises important questions as to which phenotypes observed in other transgenic SBMA mouse models are related to a polyQ expansion *versus* overexpression of the AR. Indeed, overexpression of the wild-type form of ataxin-1, another protein implicated in polyQ disease, also causes disease in mice [182].

Although many aspects of SBMA are recapitulated in transgenic models of SBMA, the knock-in model is the only tool available to SBMA researchers in which AR is expressed at precisely endogenous levels and regulated by endogenous regulatory elements. A knock-in model of SBMA was created in which a large part of exon 1 of the mouse AR gene was replaced with corresponding exon 1 sequences of the human AR containing 21Q or 113Q [183, 184]. Mice expressing AR21Q and female mice do not develop a disease phenotype, whereas male mice expressing AR113Q exhibit decreased grip strength beginning at 8 weeks and subsequently display a progressive motor phenotype [185]. High expression of the AR in the levator ani/bulbocavernosus muscles leads to urinary tract obstruction and early death in a subset of knock-in SBMA mice, but mice that survive this bottleneck live a normal lifespan [185]. Muscle pathology is apparent in these mice and ubiquitin-positive AR aggregates are present in spinal cord motor neurons [185]. Importantly, AR113Q knock-in mice display signs of androgen insensitivity, a key aspect of disease manifestation in SBMA patients that is not observed in other mouse models [185].

Altogether, there are a number of SBMA mouse models available to researchers, each with a unique set of advantages and disadvantages. However, important caveats remain in the use of any mouse model. With particular focus on the study of SBMA, it should be noted that the AR polyQ tract length in each of these models far exceeds the longest found in patients [47]. The length of the polyQ tract can have important implications in the pathology mediated by a polyQ-disease–causing protein, as has been demonstrated in HD [186]. Because polyQ tract length can affect which tissues are affected (and to what degree), caution is warranted in the interpretation of tissue-specific effects in mice expressing AR with extraphysiological polyQ tract lengths. Furthermore, although motor neuron loss was reported in the YAC-SBMA model, other models exhibit no significant motor neuron loss [8, 177, 178, 185]. This is particularly relevant in the testing of therapeutics. Postsymptomatic SBMA patients have substantial motor neuron loss [5]; thus, therapeutics that reverse motor dysfunction in mouse models may not be relevant for patients, as lost motor neurons cannot be recovered.

## Clinical Trials and Outcomes

### Leuprorelin [187–189]

The requirement for hormone in the pathogenesis of SBMA has been demonstrated in multiple animal models of the disease [175, 177, 178, 185] and is supported by the fact that females homozygous for polyQ-expanded AR display only a mild SBMA phenotype [190]. The therapeutic potential of androgen reduction was first examined in a transgenic mouse model of SBMA and 2 approaches were evaluated. Flutamide, a competitive antagonist of the AR, promoted AR nuclear localization and was unable to rescue the SBMA phenotype in mice, whereas the gonadotropin-releasing hormone (GnRH) agonist leuprorelin acetate led to a substantial rescue of motor function and reduced nuclear AR [191]. Initially, agonism of GnRH receptors by leuprorelin acetate stimulates production of LH and follicle-stimulating hormone (FSH) by the pituitary gland, which increases testosterone production [192]. However, GnRH receptors become desensitized with continuous treatment of leuprorelin, ultimately leading to reduced secretion of LH and FSH by the pituitary gland and decreased production of testosterone in the testes [192]. Treatment of SBMA patients with leuprorelin acetate yielded promising results in a phase 2 clinical trial in which 48 weeks of randomized, placebo-controlled treatment followed by an additional 96 weeks of open-label treatment significantly improved swallowing function [189]. Moreover, autopsy of 1 leuprorelin-treated patient suggested that leuprorelin may decrease the nuclear accumulation of mutant AR in motor neurons of the brainstem and spinal cord [189]. A larger, phase 3 clinical study was then conducted in which 100 SBMA patients (99 placebo controls) were treated with leuprorelin for 48 weeks and evaluated for swallowing function [187]. Contrary to the results of the smaller phase 2 study, no significant improvement in swallowing function was observed [187]. More recently, a follow-up analysis of 36 patients treated with leuprorelin for 84 months (open-label) found a slower decline in motor function than in nontreated controls, as determined by the amyotrophic lateral sclerosis (ALS) Functional Rating Scale (ALSFRS-R), the Limb Norris Score, and the Norris Bulbar Score [188]. Event-free survival was also increased in the leuprorelin-treated group [188]. Although the interpretation of these results is complicated by the lack of a placebo control, taken together, there may be a modest benefit for SBMA patients treated with leuprorelin acetate. Early intervention is likely key, as leuprorelin treatment led to a greater improvement in swallowing function in patients with a disease duration of less than 10 years [187].

### Dutasteride [4]

Although the neurologic phenotypes associated with SBMA are caused by a gain of toxic function of the mutant AR, loss of AR function causes a number of deleterious symptoms in patients [15]. Thus, there is rationale for selectively blocking the activation and nuclear translocation of the mutant AR although retaining some of the trophic effects of testosterone [193]. The type 1 and type 2 5α-reductase inhibitor dutasteride blocks the conversion of testosterone to the more potent androgen DHT and is approved for use in the USA for the treatment of benign prostatic hyperplasia (enlarged prostate) [194]. A randomized, double-blind clinical trial was conducted to assess the therapeutic potential of dutasteride in SBMA, in which 25 patients were treated with dutasteride for 24 months and compared with 25 placebo controls [4]. Neither the primary outcome measure, an assessment of muscle strength, nor any quantitative secondary measures improved as a result of Dutasteride treatment, despite the fact that, as predicted, DHT levels decreased approximately 90% in treated patients, whereas testosterone levels were unaffected [4]. These results suggest that testosterone is sufficient to activate and maintain the pathogenic state of mutant AR.

### Exercise [195]

Exercise is well-known to be beneficial to overall health, but there is rationale for caution in the use of exercise as a therapy for neuromuscular disorders, as it has been hypothesized that exercise may increase the rate of muscle atrophy in ALS. This is primarily based on 2 observations: first, that high-level athletes are at a greater risk for developing ALS [196]; and second, that when symptom onset is asymmetrical in ALS patients, it occurs disproportionately on the dominant side [197]. This finding is mirrored in SBMA patients who, when symptom onset is asymmetrical, complain of muscle weakness on their dominant side ~70% of the time [14, 30]. Having noted these concerns, thus far, clinical trials studying the effect of exercise on ALS patients have not reported negative outcomes. In fact, studies have shown that exercise significantly improves muscle strength [198, 199] and quality of life scores [199] in ALS patients. There is an additional rationale for the therapeutic potential of exercise in the treatment of SBMA, as exercise is known to increase circulating levels of insulin-like growth factor 1 (IGF-1) [200], which is protective in animal models of SBMA [125, 201]. Based on these data, a clinical study was performed in which 50 SBMA patients participated in either ‘functional exercise’ (24 patients) or stretching (control, 26 patients) for 12 weeks [195]. Overall, exercise did not cause a significant improvement in either the primary outcome measure (Adult Myopathy Assessment Tool (AMAT) score), or on any secondary measures, which included evaluations of muscle strength, balance, quality of life score, and IGF-1 levels [195]. However, when patients were subdivided into high- or low-functioning groups based on their initial AMAT scores, exercise was found to have significantly improved the AMAT scores of low-functioning patients [202]. These findings may suggest that the functional exercise routine performed by patients in this study was too light, particularly for those subdivided into the high-functioning group. The effect of high-intensity training on disease progression in SBMA is currently being investigated (clinicaltrials.gov—NCT02156141).

### BVS857 [203]

IGF-1 is known to promote growth and regeneration of skeletal muscle [204] and to protect against motor neuron death in mouse models of ALS [205, 206]. Additionally, IGF-1 signaling activates Akt which, via phosphorylation of the mutant AR at 2 Akt consensus sites (S215 and S792), decreases its ligand binding, nuclear translocation, transcriptional activation, and toxicity in cell and animal models of SBMA [124, 125, 201]. Based on the protective effects of both genetic overexpression of IGF-1 as well as injection of IGF-1 in SBMA mice, a double-blind, placebo-controlled clinical trial was conducted in which BVS857, an IGF-1 mimetic with improved pharmacological properties, was administered to 18 SBMA patients (9 placebo controls) for 12 weeks [203]. Although no serious adverse side effects were reported in the 18 patients receiving BVS857, 11 developed an immune response against BVS857 and 5 patients produced cross-reactive antibodies capable of neutralizing endogenous IGF-1 [203]. Although this immune response did not cause any observable symptoms in these patients, the detection of IGF-1 neutralizing antibodies in patients treated with BVS857 poses a serious challenge to its long-term use as a therapy for SBMA. Despite a relatively small sample size and the short treatment duration, a significant improvement in thigh muscle volume and a trend toward improved lean body mass were reported in BVS857-treated patients [203]. These outcomes indicate that there may be therapeutic potential for IGF-1 in the treatment of SBMA, although activation of the IGF-1 signaling pathway by other means may be necessary to avoid an adverse immune response.

### Creatine Monohydrate [207]

It has been previously shown that SBMA patients exhibit reduced serum creatinine levels and that there is an inverse relationship between serum creatinine levels and motor dysfunction [208]. Creatinine is synthesized intracellularly from its precursor phosphocreatine, which is taken into the cell via the creatine transporter SLC6A8 [209]. In both skeletal muscle from SBMA patients and in cultured C2C12 muscle cells expressing mutant AR, SLC6A8 protein expression is decreased, providing a possible mechanism for the observed reduction in serum creatinine in SBMA patients [171]. Additionally, it is known that slow-twitch, type 1 muscle fibers store less phosphocreatine than fast-twitch, type 2 muscle fibers [210], and that type 2 to type 1 fiber switching occurs in SBMA patients and mouse models of SBMA [132, 211, 212]. These data provided the rationale to conduct a randomized, double-blind, placebo-controlled clinical trial to explore the potential of creatine monohydrate supplementation to attenuate muscle weakening in SBMA patients. The study enrolled 45 SBMA patients who were divided evenly between 3 groups: placebo, 10 g creatine monohydrate/day, and 15 g creatine monohydrate/day [207]. Creatine monohydrate was taken orally for 8 weeks, with the primary endpoint at the conclusion of the trial being handgrip strength [207]. Secondary endpoints included respiratory and swallowing function, skeletal muscle mass, and quality of life score [207]. Although the results of this study are not currently available, there is reason for optimism as creatine supplementation has had a beneficial effect in Duchenne muscular dystrophy patients [213–215].

## SBMA—Open Questions

Given the lack of an effective treatment for SBMA following multiple clinical studies, fundamental aspects of the disease are continuing to be studied in an effort to more carefully design the next clinical trials. Indeed, new discussions regarding the role of hormone in SBMA have been driven by the fact that clinical trials aimed at suppressing hormone production in SBMA patients have had modest effects on disease progression [4, 187], despite the fact that chemical or surgical castration completely abolishes (and even reverses) disease in mouse models [177, 178, 185, 191]. It has recently been suggested that some subclinical phenotypes in SBMA mouse models may be hormone independent [216], although the authors of this study note that prenatal androgen exposure during development [217] represents an important caveat. Nevertheless, a conversation regarding the role of hormone in disease is worthwhile as efforts to knock down mutant AR mRNA in SBMA patients gain traction based on promising preclinical studies [169, 218]. The question arises: if hormone suppression has little effect on disease progression in postsymptomatic SBMA patients, why will an AR knockdown succeed?

The requirement for androgens in SBMA pathogenesis is exemplified by the fact that expression of mutant AR in heterozygous females primarily results in mild or subclinical symptoms [9, 31]. Although some heterozygous women have been reported to experience muscle weakness (possibly correlated with skewed X-chromosome inactivation of the normal AR gene [219]), evaluation of 2 homozygous women revealed only mild muscle cramping and tremor [190]. This case study clearly indicates that hormone is required for manifestation of the human disease: although it is notable that homozygous females are spared from SBMA, removing hormone from male patients has little effect on disease progression, albeit with treatment starting well after disease onset.

Another intriguing case study may hold additional insights. This report describes a male-to-female transgender SBMA patient who developed full disease phenotype despite 15 years of treatment with an anti-androgen (leading to undetectable levels of androgens) [220]. Although this patient noted gynecomastia before starting therapy, she did not develop muscle weakness until 4 years after anti-androgen treatment and was not diagnosed with SBMA until 6 years after beginning treatment [220]. In this case, the anti-androgen used by the patient, spironolactone, caused some AR nuclear localization, aggregation, and transcriptional activation in an SBMA cell model as well as slight, but significant toxicity in a *Drosophila* model [220]. Although the effect of spironolactone in causing these phenotypes was substantially reduced compared to DHT, these findings do provide 1 explanation for the development of SBMA in this patient. Nonetheless, it is notable that disease in this patient was not slowed relative to her brother despite presymptomatic anti-androgen treatment. Muscle weakness was noted by the patient (AR49Q) at age 29 and her brother (AR50Q) at age 34 [220].

These two case studies could indicate that there is a developmental aspect to SBMA, wherein androgen exposure that occurs in males prenatally or during puberty sets the disease in motion before symptoms appear. Additional changes to the cellular environment that occur with aging may ultimately lead to motor neuron death later in life, even if circulating androgen levels are reduced. Hormone is clearly a requirement for disease, as even homozygous females exhibit only mild symptoms; however, it is also clear that reducing androgen levels in postsymptomatic patients has limited effects on disease progression. Although 14 years of androgen deprivation slowed disease progression of 1 SBMA patient relative to historical data [221], it should be noted that this patient began androgen deprivation (by treatment with leuprorelin acetate) only 6 months after symptom onset [221]. Thus, the slow disease progression of this patient highlights the importance of early intervention as much as the potential benefits of long-term androgen deprivation.

Additionally, the question of which cell type(s) drive disease is of vital importance for the next generation of therapies. Some studies have suggested that peripheral knockdown of the AR is sufficient to rescue disease phenotypes in SBMA mouse models [8, 169]. Should these findings translate to patients, it would simplify drug delivery and spare androgen receptor function in the CNS [222]. However, there is also evidence that expression of the AR in the CNS drives disease [178, 218]. Additionally, SBMA patients suffer from sensory neuropathies, further suggesting a primary role for CNS AR in disease.

Thus far, all of the studies conducted in SBMA mouse models suffer from the caveat that, in order to drive an observable behavioral phenotype, AR with a supraphysiological polyQ tract length is expressed. In HD, long polyQ tracts are associated with juvenile onset HD, which is characterized by dysfunction of different brain regions (notably, the cerebellum) than adult onset HD [186]. Given that polyQ tract length can affect which cell types are disrupted by the mutant protein (and to what degree), caution is therefore warranted in the interpretation of tissue-specific effects in these SBMA mouse models. It is well-established that toxicity in motor neurons can drive muscle pathology and that toxicity in muscle can drive motor neuron pathology. Consequently, similar phenotypes could be achieved in SBMA mice regardless of whether muscle or motor neurons drive disease. The goal, however, is to determine which cell types are critical for disease in human SBMA patients. This question was addressed in HD by utilizing an allelic series of knock-in mice to gather transcriptomics data on various tissues in response to huntingtin with a range of polyQ tract lengths [223]. Perhaps a similar approach in an SBMA mouse model could shed light on how polyQ tract length impacts the cell types affected by the mutant AR. Given that polyQ tract length inversely correlates with disease onset, identifying the genes that change most in response to a longer polyQ tract could be important, as these genes must contribute to the disease. Moreover, determining if transcriptomic/proteomic phenotypes at a physiologically relevant polyQ tract length occur first in motor neurons or muscle could guide efforts to effectively deliver SBMA therapies moving forward.

Related to the question of the tissue involved in disease initiation, the extent to which tissue-specific somatic instability of the expanded CAG tract of mutant AR contributes to disease remains to be determined. Somatic mosaicism is primarily observed in cardiac and skeletal muscle of SBMA patients—tissues with high levels of AR expression [224]. Additionally, a higher CAG mosaicism index in SBMA patient blood inversely correlated with age of onset in a study of 46 SBMA patients, although statistical significance was not reached for other clinical features of disease [30]. Clarifying the role of somatic instability in SBMA pathogenesis could add to our understanding of why specific cells/cell types are vulnerable to polyQ-expanded AR.

It remains to be determined which direction(s) of study will ultimately lead to a treatment for SBMA, but until effective treatments exist, further exploration in all directions is imperative. The studies summarized here reflect broad efforts to advance our understanding of SBMA on multiple fronts, with the ultimate goal of achieving a clinically meaningful outcome in patients.
